# The ‘when’ and ‘where’ of semantic coding in the anterior temporal lobe: Temporal representational similarity analysis of electrocorticogram data

**DOI:** 10.1016/j.cortex.2016.02.015

**Published:** 2016-06

**Authors:** Y. Chen, A. Shimotake, R. Matsumoto, T. Kunieda, T. Kikuchi, S. Miyamoto, H. Fukuyama, R. Takahashi, A. Ikeda, M.A. Lambon Ralph

**Affiliations:** a Neuroscience and Aphasia Research Unit, School of Psychological Sciences, University of Manchester, Manchester, UK; b Department of Neurology, Graduate School of Medicine, Kyoto University, Japan; c Department of Epilepsy, Movement Disorders and Physiology, Graduate School of Medicine, Kyoto University, Japan; d Department of Neurosurgery, Graduate School of Medicine, Kyoto University, Japan; e Human Brain Research Center, Graduate School of Medicine, Kyoto University, Japan

**Keywords:** Semantic representation, Anterior temporal lobe, Representational similarity, Multi-voxel pattern analysis, ECoG, electrocorticogram, RSA, representational similarity analysis, ATL, anterior temporal lobe

## Abstract

Electrocorticograms (ECoG) provide a unique opportunity to monitor neural activity directly at the cortical surface. Ten patients with subdural electrodes covering ventral and lateral anterior temporal regions (ATL) performed a picture naming task. Temporal representational similarity analysis (RSA) was used, for the first time, to compare spatio-temporal neural patterns from the ATL surface with pre-defined theoretical models. The results indicate that the neural activity in the ventral subregion of the ATL codes semantic representations from 250 msec after picture onset. The observed activation similarity was not related to the visual similarity of the pictures or the phonological similarity of their names. In keeping with convergent evidence for the importance of the ATL in semantic processing, these results provide the first direct evidence of semantic coding from the surface of the ventral ATL and its time-course.

## 1 Introduction

Understanding the meanings of objects and words is an essential skill for everyday living and communication. Thus, semantic impairments after neurological damage significantly compromise quality of life. Semantic information must be both represented in the brain and readily accessible when needed. Exactly where in the brain semantic coding for individual concepts takes place and how semantic retrieval unfolds over time are still debated (Binder et al., 2009, Lambon Ralph, 2014). We directly address these questions by applying, for the first time, representational similarity analysis (RSA) (Kriegeskorte, Mur, & Bandettini, 2008) to high temporal-resolution electrocorticogram (ECoG) data collected whilst patients were engaged in a picture naming task (a simple task that necessitates semantic retrieval). RSA is a special type of multi-voxel pattern analysis (MVPA). Unlike other MVPA methods that train a classifier to distinguish between two categories, RSA formally compares the similarities coded in observed neural activity against various hypothesised representations. For example, the pattern of activation across a set of voxels (or, in the case of our study, electrodes) is recorded for a collection of stimuli. All pairwise similarities are computed for these observed neural activations. The experimenter then tests how well these observed similarity patterns match hypothesised models (e.g., does the pattern mimic semantic similarities, visual similarities, etc.) Thus, RSA offers a method to explore representational coding down to the level of individual items rather than coding membership of two broad categories.

Classical models of conceptualisation and contemporary ‘strong’ embodied theories of semantic representation argued that concepts reflect the joint activation of verbal and sensorimotor information that is distributed across the cortex (Eggert, 1977, Gainotti, 2015, Meteyard et al., 2012). Damasio and colleagues (Damasio et al., 1996, Damasio et al., 2004, Tranel et al., 1997) proposed that this distributed collection of information is brought together through dedicated multimodal hubs or ‘convergence zones’. Further development of these ideas, informed by neuropsychological and computational explorations, gave rise to the implemented ‘hub-and-spoke’ model which was able to demonstrate how a transmodal hub could interact with modality-specific sources of information in order to give rise to coherent, generalisable concepts and, after damage, was able to mimic patients' semantic impairments (Lambon Ralph et al., 2007, Rogers et al., 2004). There is growing evidence that regions within the anterior temporal lobe (ATL) play a critical role as a core semantic representational hub (for important alternative considerations of left versus right ATL function, see: Drane et al., 2013, Gainotti, 2015, Rice et al., 2015a, Rice et al., 2015b, Rice et al., 2015b). An important initial and strong source of evidence for this hypothesis came from patients with semantic dementia whose progressive ATL-centred atrophy leads to selective semantic degradation without generalised cognitive impairment (Lambon Ralph et al., 2010, Patterson et al., 2007, Warrington, 1975). The degradation of semantic information is multi-modal. Patients cannot activate meaning from any modality, whether they are seeing an object, hearing the sound of the object or its spoken name (Bozeat, Lambon Ralph, Patterson, Garrard, & Hodges, 2000). This suggests a genuine degradation of semantic information rather than failure of retrieval. The anterior temporal lobe also exhibits greater metabolic activity when healthy participants are engaged in semantic tasks compared with control non-semantic tasks in neuroimaging studies (Binney et al., 2010, Marinkovic et al., 2003a, Vandenberghe et al., 1996, Visser et al., 2010). Furthermore, when the ATL is stimulated using transcranial magnetic stimulation (TMS), participants suffer from a temporary drop in semantic performance across various object categories and tasks (Pobric et al., 2007, Pobric et al., 2010), mirroring the chronic deficit observed in semantic dementia patients. Psychophysiological studies have also revealed anterior temporal area as a potential source for semantic priming effects (Geukes et al., 2013, Lau et al., 2013). A recent study using combined electroencephalogram (EEG)/magnetoencephalography (MEG) for source localisation observed greater responses in ATL in semantic decision tasks as compared to silent reading (Chen, Davis, Pulvermuller, & Hauk, 2013).

Despite the well-established importance of the ATL region in supporting semantic task performance, the questions of which subregions are the most important and how rapidly this information is activated, are still unanswered. It is possible that the ATL supports a function necessary in semantic tasks but does not code semantic representations per se, or that it provides linkage to other brain regions that actually store meanings (Damasio et al., 2004). Likewise there is very little information on the time course of these ATL subregions. Accordingly, both the location and the timing of the semantic information arising in these key ATL areas were the focus of this study.

Recent multivariate pattern analysis functional magnetic resonance imaging (fMRI) studies have investigated which regional activity correlates with semantic structure (Bruffaerts et al., 2013b, Clarke and Tyler, 2014, Fairhall and Caramazza, 2013, Peelen and Caramazza, 2012). Although two investigations used region of interest (ROI) analyses and a small set of concepts to probe ATL semantic contributions (Coutanche and Thompson-Schill, 2014, Peelen and Caramazza, 2012), the results across studies have been largely mixed and inconsistent, which may be due to a number of factors including variations in task, concepts probed and the MVPA methods, in addition to the well-known fMRI signal dropout and distortion problems associated with the ventral ATL and other regions (Devlin et al., 2000, Visser et al., 2010).

An ideal method would be capable not only of pinpointing the brain region(s) involved (ultimately measuring the brain activity directly rather than indirectly through fMRI, MEG, etc.) but also of capturing the temporal dynamics of semantic processing. The combination of precise spatial and temporal information is hard to achieve with fMRI (good spatial but poor temporal resolution) or MEG/EEG (good temporal but poor spatial resolution). ECoG [in the form of evoked local field potentials (LFP) measured at the cortical surface through implanted grid electrodes] meets both methodological requirements. Consistent with the neuropsychology and cognitive neuroscience studies noted above, previous electrode studies have confirmed the involvement of ventral and lateral ATL regions in semantic tasks. Previous studies demonstrated evoked LFPs in this region for picture naming and transient impairment after direct electrical cortical stimulation (Lüders et al., 1986, Lüders et al., 1991, Nobre et al., 1994, Shimotake et al., 2015). Contemporary investigations have shown evoked responses for naming irrespective of input modality and that the ventrolateral ATL is crucial for a variety of verbal and nonverbal semantic tasks (Abel et al., 2015, Shimotake et al., 2015). However, none of these studies investigated semantic coding in these regions or its time-course.

Establishing both the critical regions for semantic representation and the timing of their contribution are important not only for understanding the neural basis of semantic memory but also for clinical neuroscience. The ATL regions are compromised in a range of neurodegenerative disorders (e.g., frontotemporal dementia, Alzheimer's disease, etc.) and some types of acute neurological damage (e.g., head injury). In addition, it is sometimes necessary to resect some or all of this region, either for tumour resection or to treat intractable temporal lobe epilepsy. Indeed, ever since the seminal studies of Lüders and colleagues, neurosurgeons have been aware of the potential role of ventral ATL regions in language processing (the “basal temporal language area”) and direct electrical stimulation (DES: Duffau, 2005, Duffau, 2015) is now commonly used during resection procedures to assess for potential language-related eloquent tissue in this region. When it is clinically possible to avoid resection of the ventral ATL (by using a sub-temporal approach), patients have better long-term recovery of verbal memory (Mikuni et al., 2006). Our current study was also designed, therefore, to test a key hypothesis raised by previous fMRI and intracranial electrode studies (as proposed by Shimotake et al., 2015) that the contribution of the basal temporal language region to verbal and nonverbal processing is rooted in its more primary role in the representation of semantic concepts.

We tackled these important clinical and basic science questions through two innovations. We applied RSA (Kriegeskorte et al., 2008) for the first time to ECoG data collected on 100 concepts. Secondly, given the rarity of this neurosurgical procedure, ECoG data are traditionally collected and reported for individual patients, which makes it hard to compare between them or to functional neuroimaging in healthy participants, where results are analysed at the group level. In this study, therefore, we also utilised new methods to combine data from a case-series of ten patients (accumulated over a two-year period) to reveal the statistically-significant group-wide consistent results. This allowed us to establish: (a) which temporal lobe regions reliably generate LFP that correlate with item-specific semantic representation; and (b) when in the evoked response this semantic coding is present.

## 2 Materials and methods

### 2.1 Participants

Ten patients with intractable partial epilepsy (eight) or brain tumour (two, one associated with intractable partial epilepsy) participated in this study. Background clinical information about each patient is summarized in Table 1. Subdural electrode implantation was performed in the left (eight) or right (two) hemisphere for presurgical evaluation (mean 83 electrodes, range 56–107 electrodes/patient). 6–30 electrodes (mean 20 electrodes) covered the ventral ATL in each patient. The subdural electrodes were constructed of platinum with an inter-electrode distance of 1 cm and recording diameter of 2.3 mm (ADTECH, WI). ECoG recording with subdural electrodes revealed that all epilepsy patients had seizure onset zone outside the anterior fusiform region, except one patient for whom it was not possible to localize the core seizure onset region. The study was approved by the ethics committee of the Kyoto University Graduate School of Medicine (No. C533). Participants all gave written information consent to participate in the study.

### 2.2 Stimuli and procedure

One hundred line drawings (50 living and 50 nonliving items) were obtained from previous norming studies (Morrison et al., 1997, Snodgrass and Vanderwart, 1980). A complete list of all items can be found in Table S1 (Supplementary materials). Living and nonliving stimuli were matched on age of acquisition, visual complexity, familiarity and word frequency. Independent-sample *t*-tests did not reveal any significant differences between living and nonliving items for any of these variables.

Participants were presented with stimuli on a PC screen and asked to name each item as quickly and accurately as possible. All stimuli were presented once in a random order in each session and repeated over four sessions in the entire experiment. The responses of participants were monitored by video recording. Each trial was time-locked to the picture onset using in-house MATLAB scripts (version 2010a, Mathworks, Natick, MA). Stimuli were presented for 5 sec each (the patients' average naming time was 1190 msec) and each session lasted 8 min 20 sec. Participants' responses and eye fixation were monitored by video recording.

### 2.3 Data preprocessing

Data preprocessing was performed in MATLAB. Raw data were recorded at sampling rate of 1000 Hz. The raw data from the target subdural electrodes for the subsequent analysis were measured in reference to the electrode beneath the galea aponeurotica in six patients (Patients 4–8 and 10) and to the scalp electrode on the mastoid process contralateral to the side of electrode implantation in four patients (Patient 1–3 and 9). Data were divided into epochs that started from 200 msec before to 1000 msec after each picture onset. Baseline correction was performed by subtracting the mean pre-stimulus baseline amplitude (200 msec before picture onset) from all data points in the epochs. Trials with greater than ±500 μV maximum amplitude were considered rejected as artifacts. Visual inspection of all raw trials was conducted to reject any further trials contaminated by artifacts, including canonical interictal epileptiform discharges. The mean waveform for each stimulus was computed across repetitions.

### 2.4 Data analysis

Representation similarity analysis was combined with a spatio-temporal searchlight (Su, Fonteneau, Marslen-Wilson, & Kriegeskorte, 2012). The key idea is to perform RSA analysis on a small proportion of the whole spatio-temporal data and then repeat it iteratively for the whole data set by moving the searchlight across space and time.

At the single subject level, data for each item and each electrode were first averaged over sessions. A searchlight contained data from one electrode and its neighbouring electrodes across a 100-msec time window. These data were converted into a long vector for each item and used to compute correlational distance between each pair of stimuli. This computation resulted in a neural dissimilarity matrix with the number of rows and columns equal to the number of stimuli. This neural matrix was compared with the theoretical models (visual, phonological and semantic models – specified below). As the bottom-left and top-right entries of these matrices were identical, the bottom-left half of both the neural and theoretical matrices, excluding the diagonal entries which were all 0, were extracted and converted into vectors. The spearman correlation coefficient between these vectors reflected the similarity between the neural matrix and each of the theoretical matrices. The searchlight was then moved across space and time iteratively to compare neural and various theoretical models. The correlation coefficient was stored at central voxel location of each searchlight.

**Fig. 1:**
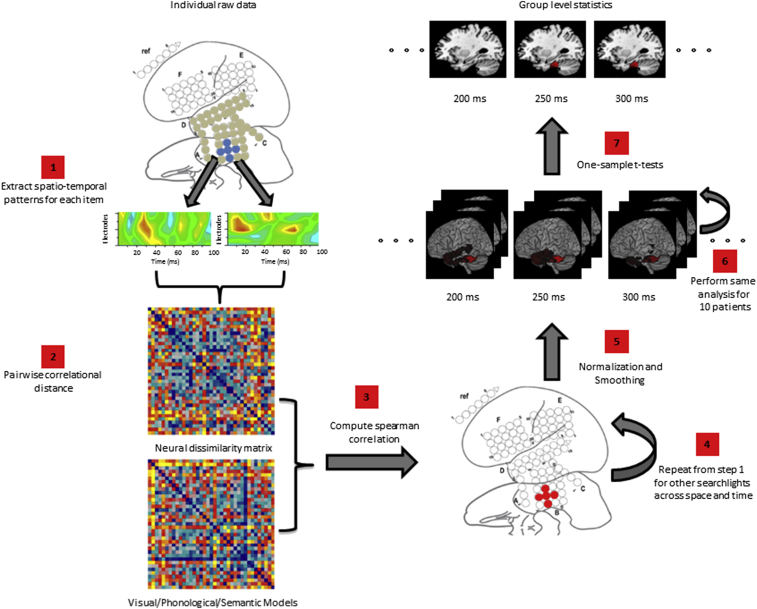
Analysis pipeline. 1) The brain figure summarises the grid positions for one patient (open circles). The number, shape and placement of grids varied across the ten patients. All had grids inserted into the ventral and lateral temporal area (filled circles) so data were considered from these sites in all cases. The spatio-temporal pattern was extracted for each item at each temporal electrode (averaging the electrode and its immediate neighbours – blue filled circles). 2) A neural dissimilarity matrix was computed by correlating the extracted spatiotemporal patterns between all item pairings. 3) The resultant neural dissimilarity matrix was compared to the theoretical representational models (visual/phonological/semantic). 4) The correlation test statistic for each electrode site was saved; steps 1–4 were repeated for all available temporal electrodes and all time windows under consideration (either whole epoch or divided into time-windows). 5) The participant's brain was normalised into MNI space and the searchlight results for each electrode were rendered into the corresponding voxels. 6) Steps 1–5 were repeated for all ten patients. 7) One sample *t*-tests were computed at each time window to obtain group-level statistics. Random field theory was used to correct for multiple comparisons.

Magnetization-prepared rapid gradient-echo (MPRAGE) volumetric scan was performed before and after implantation of subdural electrodes as a part of presurgical evaluations. In the volumetric scan taken after implantation, the location of each electrode was identified on the 2D slices using its signal void due to the property of platinum alloy (Matsumoto et al., 2004). Electrodes were non-linearly co-registered to the patient MRI (MPRAGE) taken before implantation, and then to MNI standard space (ICBM-152) using FNIRT (www.fmrib.ox.ac.uk/fsl/fnirt/). The native coordinates of all the electrodes for all patients were morphed into MNI space and resampled into 2 mm isotropic voxels (Matsumoto et al., 2011). Voxels that were within the radius of a searchlight (i.e., 10 mm or the distance between neighbouring electrodes) were rendered with the amplitude of their nearest searchlight peak. If a voxel was within the coverage of more than one searchlight, it was assigned the average amplitude of all these searchlights. Single subject correlation coefficient maps were subjected to random effects analysis (RFX) and group level interpretation using SPM8. See Fig. 1 for a complete analysis pipeline.

Our statistical analysis contained three parts. First, to provide an overview of which spatial regions are correlated with various models across the whole epoch, spearman correlation coefficient maps for all time windows were averaged and subject to SPM RFX. Second, a SPM RFX was conducted in each time window to establish when there was a significant correlation with any theoretical model at the group level. The purpose of this analysis was to determine semantic effects over time. Third, we also performed a targeted analysis of the ventral anterior temporal lobe, which plays a pivotal role in general semantic processing, as shown by previous studies (Binney et al., 2010, Visser et al., 2010). Correlation coefficients for each theoretical model were averaged across all voxels within this ROI and subjected to analysis for each time window. Two types of analysis were performed. One-sample *t*-tests were conducted to test if the correlation coefficient for each model was significantly above 0. A repeated-measure analysis of variance (ANOVA) was also performed to test if there were any significant differences between correlation coefficients of different models. If the ANOVA was found to be significant, post-hoc pair-sample *t*-tests were conducted to find out which set of models have significantly different correlation coefficients. Greenhouse-Geisser correction was applied if sphericity was violated. A statistical threshold of *p* < .05 FWE cluster-level corrected was applied unless otherwise stated.

**Fig. 2:**
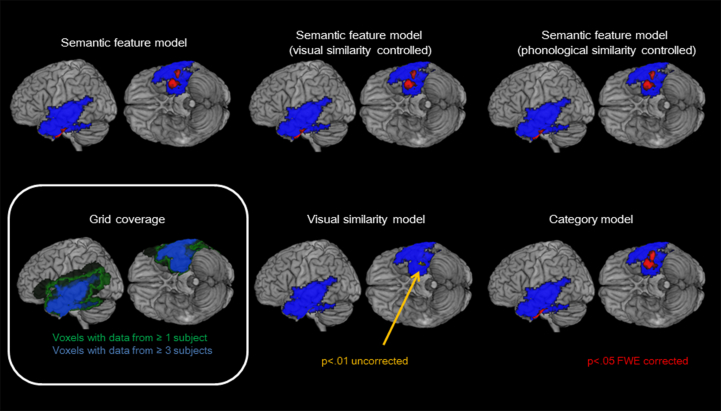
Panel A – grid coverage (blue overlay = brain voxels with data from ≥3 patients; green overlay ≥1 patients). Panel B – RSA for the visual similarity model. Panel C– RSA for the semantic category model. Panel D – RSA for the semantic feature model alone and with visual or phonological similarity partialled out. Panels B–D: blue overlay as per Panel A; red denotes regions that were significant at *p* < .05 FWE cluster corrected; yellow – denotes the weak effect for the visual similarity mode (*p* < .01 voxel uncorrected). These RSA analyses were based on the entire trail epoch – the temporal RSA variation is shown in Fig. 3.

As the number and placement of electrode grids vary across participants, we decided to focus our analysis on the entire temporal lobe because electrode coverage was most consistent in this area. In order to reduce type II error and avoid unnecessary multiple comparison corrections in brain regions that do not even have embedded subdural electrodes, a brain mask was created to restrict group level analysis within areas that have data from at least three patients (see Fig. 2).

Two patients had subdural electrodes embedded in the right hemisphere. Their MNI x-coordinates were flipped into the left hemisphere before group level analysis was performed. This transformation was performed to increase statistical power by including as many participants as possible. A separate analysis was conducted, removing the two right hemisphere participants. The results of the study were unchanged.

As well as testing the statistical reliability of the ECoG results across participants at the group level (as per a standard group functional neuroimaging study and a first for ECoG studies), we also explored the individual level results given that there was some variability in their aetiology and neurological histories (see Table 1). Specifically, all the voxels across all time windows for each subject were extracted. Those with top 20% largest correlation coefficients for semantic feature model were plotted (see Fig. 4).

### 2.5 Models

Picture naming contains a series of processes. It requires visual processing, retrieving semantics from the seen object and articulating the phonological output (Levelt, 1992; Laine & Martin, 1996). We therefore tailored our theoretical models to capture these key processes by computing visual, semantic and phonological relationship between the stimuli. First, chamfer matching (Borgefors, 1988) was used to capture visual similarity between the pictures. It calculates the minimum number of pixel movements required to transform one line drawing to another. The size of an image and its position in the visual space can be arbitrary so for each pair of stimuli we calculated a large number of visual dissimilarity scores by iteratively changing the scaling of one of the images and moving it in all possible translation directions in 2D space. From these many comparisons, we took the minimum score (i.e., maximising the similarity value). Fig. S1 provides a graphic illustration of the chamfer matching calculation. Second, phonological similarity model was generated by computing the Japanese mora and phoneme overlap between the names of each pair of stimuli. As mora overlap (i.e., two different words both contain one or more same moras) essentially means that all the two sets of phonemes were matched in both phoneme identity and order, it should play a more important role in overall phonological similarity than shared phonemes only. Accordingly, we computed a weighted average of the percentage of mora and phoneme overlaps between stimuli pair, by assigning a weight of 2 to mora and a weight of 1 to phonemes. Finally, to capture semantic dissimilarity between stimuli, each stimulus was scored using binary feature lists from a recent behavioural study (Dilkina & Lambon Ralph, 2013). The features consisted of three types: perceptual (e.g., X is red), functional (e.g., X is hand held) and encyclopaedic (e.g., X is tropical). Features were binary so that an item can either have a feature or not. In this way, all stimuli could be represented by feature vectors of equal length. Semantic dissimilarity was the pairwise correlational distance between these feature vectors. Furthermore, a simple living/nonliving category dissimilarity model was used as an additional semantic model, which assumed within-category difference of 0 (for both living and nonliving) and cross-category difference of 1 (i.e., items from living versus those from nonliving categories).

## 3 Results

The mean behavioural response time for the picture naming task was 1190 msec (SD = 554 msec). No significant difference was found between response time towards living and nonliving items across participants (*t* < 2).

### 3.1 Activity patterns in ventral ATL distinguish between semantic representations

Considering the entire trial epoch, the first analysis revealed that visual and semantic feature models exhibited LFP correlation with different temporal lobe regions. The ventral anterior temporal lobe (anterior Brodmann area 20) showed strong significant correlation with the semantic feature model, as shown in Fig. 2 and Table 2. The visual similarity model correlated with the mid-ventral temporal area, towards the posterior portion of the grid coverage, only at lenient threshold (*p* < .01 uncorrected). Phonological similarity did not show any significant correlation within the grid coverage.

**Fig. 3:**
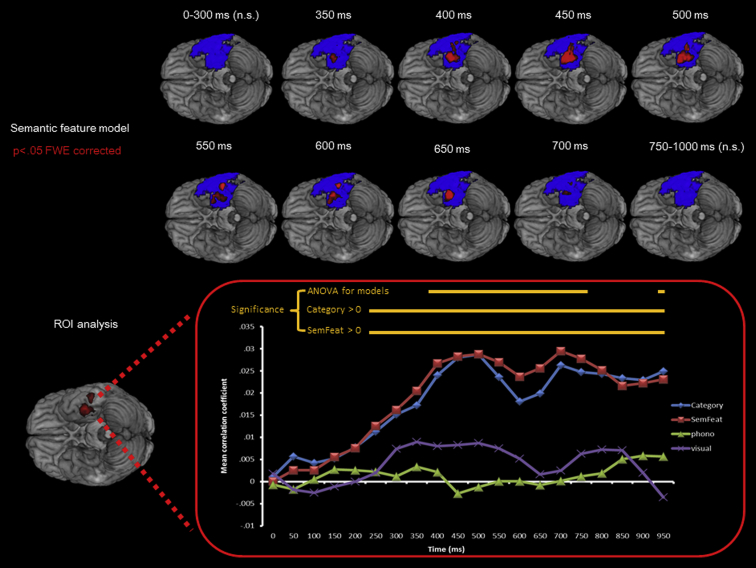
Top panel: Blue overlay denotes grid coverage. Red overlay indicates regions that showed significant correlation with the semantic feature model between 350 msec and 700 msec. The cluster at 350 msec and 700 msec was marginally significant (*p* < .08 cluster corrected) and all others survived *p* < .05 FWE cluster-correction. There were no significant correlations between 0 and 350 msec. Bottom panel: a region of interest (red) was defined as it was significant in all pictures > pre-stimulus onset baseline univariate analysis contrast. Correlation coefficients for the different models are plotted over time (see main text for description). The yellow horizontal bars denote the time-points at which each model was significant (see main text for description).

To replicate the result with a different method of coding semantic structure, a separate correlation analysis using a living/nonliving category (rather than distributed feature) model was conducted. The results were very similar to the semantic feature model (Table 2 and Fig. 3). Further analyses were conducted, therefore, with semantic feature model only.

Previous studies have shown that apparent semantic coding can reflect, instead, low level visual similarities (Bruffaerts et al., 2013a, Rice et al., 2014). Thus to test for this possibility and, by extension, any confound with phonological similarity (given that the participants generated the picture names), a partial correlation analysis between semantic feature model and neural activity patterns were conducted while visual or phonological similarities were controlled. The results revealed that the semantic feature model still exhibited the same strong correlation and was minimally affected by the inclusion of visual/phonological similarities, suggesting a genuine semantic effect.

For an initial investigation of the spatio-temporal dynamics (see Fig. 3), random-effects analysis was conducted for semantic feature correlation coefficients in a moving 100 msec time window. Considering the entire area covered by the grid electrodes, the results revealed that semantic correlations approached significance at 350 msec (*p* < .07 cluster corrected) and were robust from 400 msec to 700 msec. In comparison, visual or phonological similarity did not show any significant correlation with any temporal lobe regions in any time windows.

### 3.2 Detailed exploration of the vATL region's characteristics

As noted above, only one temporal lobe region (the vATL) was identified as exhibiting a significant correlation with the semantic feature model when considering the entire epoch as a whole. In a subsequent analysis, therefore, we conducted a more precise examination of this specific region by exploring its time-course for the semantic, visual and phonological models.

As shown in Fig. 3 (lower panel), both semantic feature and category models exhibited increasing correlation with the neural patterns in the ventral ATL, peaking at 500 msec and this strong correlation was sustained over the rest of the epoch. The correlation coefficients became statistically significant from 250 msec [category: *t*(9) = 2.31, *p* < .05; semantic features: *t*(9) = 2.38, *p* < .05]. In contrast, phonological similarity was not correlated at any time point and visual similarity was only approached significance between 450 and 500 msec [*t*(9) > 2.07, *p* < .07].

ANOVA was conducted for each time window to compare correlation coefficients for the four models. The analysis revealed a significant difference in correlation coefficients between the four models from 400 to 750 msec as well as at 950 msec [all *F*(3,27) > 4.43, *p* < .05] (Fig. 3). Post-hoc paired-sample *t*-tests conducted on these time windows confirmed that both semantic feature and category model had significantly larger correlation coefficients than the phonological and the visual model [both *t*(9) > 2.48, *p* < .05] between 400 and 550 msec. Between 600 and 750 msec, similar patterns were seen (i.e., semantic feature, category > visual and phonological) with a few exceptions [600–650 msec category > phonological: *t*(9) > 1.9, *p* < .1; 700–750 msec category > visual: *t*(9) > 1.8, *p* < .1; 750 msec semantic feature > visual: *t*(9) = 2.02, *p* < .08]. At 950 msec, both category and semantic feature models had significantly larger correlation coefficients than the visual model only [*t*(9) > 2.6, *p* < .05].

**Fig. 4:**
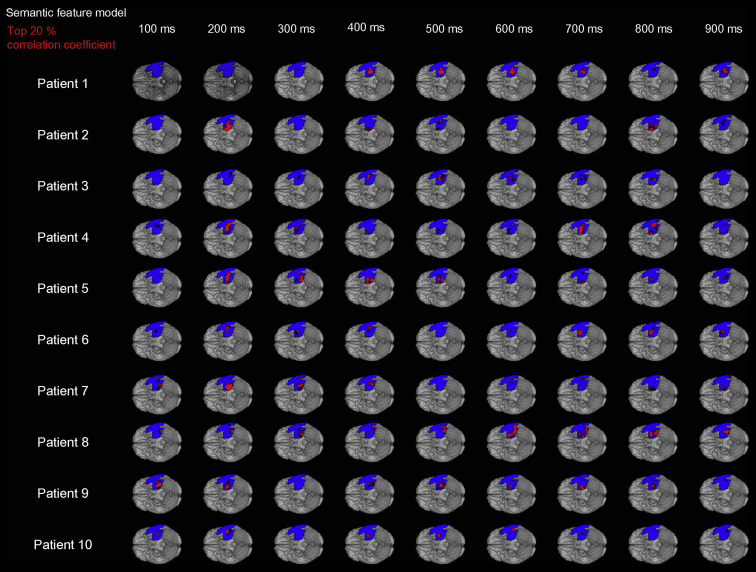
Individual participants' data. Blue overlay denotes grid coverage. Red overlay indicates regions that showed top 20% highest correlation coefficients for semantic feature model across all time windows for each patient.

To assess the potential clinical application of this new method and the consistency of the results across participants (given that there was inevitably variation in aetiology and neurological history), it is useful to check the individual patient results. As depicted in Fig. 4, the results showed that the vast majority of patients showed a response pattern like the group level data. Of course, the individual data are not exactly copy of the group average. There were some individual variations particularly of the time point at which the observed activation similarity and the semantic model became correlated. This could reflect meaningful individual differences in the onset time of semantically-coded activations in the vATL and/or it could be that the strength of the measured activations is inevitably more variable and noisier at the level of individual data. This is also true of individual functional neuroimaging data in neurologically-intact participants which, following standard statistical logic and the theorem of central tendency, is the reason why data are analysed at the group level (second level analysis) – which we have done first time with grid electrode data in this study.

## 4 Discussion

By applying temporal RSA to ECoG data, this study showed that, within the temporal lobe (where there was consistent coverage of grid electrodes across patients), naming LFPs were correlated with semantic, rather than phonological or visual structure. Secondly, these temporally-fine data indicated that this semantic coding becomes active at least by 250 msec post stimulus onset and remains throughout the remainder of the trial. Each of these key findings is discussed in a little more detail below.

### 4.1 ATL is a hub in the network for semantic representation

Existing evidence based on neuroimaging, psychophysiological and patient studies have all converged on the fact that ATL regions are necessary for multimodal semantic task performance (Binney et al., 2012, Lambon Ralph et al., 2010, Lau et al., 2013, Pobric et al., 2010). The current study goes further by providing direct evidence that the ventral ATL is a hub for semantic representation per se. This result is consistent with hypotheses from implemented computational models of semantics (Rogers et al., 2004, Schapiro et al., 2013), which propose that coherent conceptual representations are formed from the interaction of a bilateral transmodal (ATL) representation hub with various modality-specific sources of information coded in distributed secondary association cortices. This bilateral ‘hub-and-spoke’ representational framework permits key characteristics of semantic memory to emerge, including the extraction of stable representations from our rich multimodal, time- and context-varying experiences, the formation of coherent semantic representations which link in complex non-linear ways to specific features and associates, as well as computing semantic rather than superficial generalisations (Lambon Ralph, 2014, Lambon Ralph et al., 2010, Rogers et al., 2004).

It is important to be clear that we are not arguing that ATL is the only region involved in representing or processing semantics. First, as noted above, it would appear that semantic representations require the joint action of the ATL hub and the modality-specific sources of information (Lambon Ralph, 2014), made possible and efficient through local and long-range white-matter connectivity (Binney et al., 2012, Ding et al., 2009, Pascual et al., 2013). Indeed, previous seminal studies have demonstrated that these ‘spoke’ regions are activated when semantic decisions require access to the information that is experienced in that modality (Martin, 2007), and semantic performance becomes less efficient when either a dual-task interferes with modality-specific processing (Barsalou, 2008) or the spoke region is stimulated (Pobric et al., 2010). Secondly, rather than coding semantic knowledge, other tertiary cortical areas are important for manipulating, gating and suppressing different aspects of our rich semantic database in order to generate task- and context-appropriate verbal and nonverbal behaviours (Jefferies and Lambon Ralph, 2006, Noonan et al., 2013, Thompson-Schill et al., 1998, Whitney et al., 2011). Finally, it is possible that other non-temporal regions act as additional representational hubs (Binder & Desai, 2011), which this study could not evaluate given that they fall outside of the grid electrode coverage.

Secondly, we note here that in long-term conditions, the contribution of different regions to a higher cognitive function can change or shift over time – potentially changing the inferences that can be made about the normal localization of function (Duffau, 2015, Thiel et al., 2001). In the context of the current study, the previous investigation by Shimotake et al. (2015) is highly pertinent. In order to explore this possibility, Shimotake and colleagues conducted a direct comparison between the location of the critical grid site for semantic processing in the patients, and the peak activations obtained from neurologically-intact participants when undertaking various verbal and nonverbal tasks. Shimotake et al. found that the two vATL sites were remarkably similar and are the same area highlighted in the present study. This strongly suggests that this ventral ATL region remains as a critical semantic region in the patients. Of course, it is possible that other regions within the semantic network change or alter their function to support patients' semantic performance – which is not possible for us to assess in the present investigation but could be explored in future fMRI studies.

### 4.2 Timing of the vATL semantic coding

The rise of semantic coding in the vATL LFPs from 250 msec is consistent with other methods that have probed the ATL time-course for semantic processing. This includes the ATL convergence of auditory and visual semantic processing observed in MEG (Marinkovic et al., 2003), semantic priming and category differences in multimodal imaging and depth electrode studies (Chan et al., 2011, Lau et al., 2013, Nobre and McCarthy, 1995, Rossell et al., 2003). One recent MEG study found an earlier (around 100 msec) vATL effect (Clarke, Taylor, Devereux, Randall, & Tyler, 2013); specifically, an enhanced event-related regression coefficient for items with a relatively greater ratio of shared to distinctive semantic features. Future research is needed to relate these regression measures to the full coding of individual concepts (as probed in the current study). One possibility, as suggested by Clarke et al., is that very general aspects of meaning are activated in the vATL from very early time-points (presumably reflecting the initial feedforward visual input) whilst a fuller, individuated conceptual representation gradually emerges later. This hypothesis is consistent with the hub-and-spoke computational models of semantic representation; initial input to the hub layer very quickly drives apart the activation patterns of items from unrelated domains, but it takes longer for multimodal reverberation within and between the hub and spokes to settle the model into item-specific semantic representations (Rogers & Patterson, 2007).

### 4.3 Clinical implications

The implantation of subdural electrodes is used in some neurosurgical patients in order to assess the focus and nature of seizures directly, as well as map eloquent areas for language, motor and other functions. By combining this form of electrophysiology with active tasks (ECoG), it is possible to explore the cortical evoked responses across different tasks and over time. The present study demonstrates that important additional information can be extracted from these same data by utilizing RSA. This and related analysis methods (Haxby, Connolly, & Guntupalli, 2014), allow investigation of the type or form of information that is coded in specific brain regions. For the reasons set out above, in this study we assessed the potential semantic, visual and phonological representation of the ventral and lateral temporal regions. It is, of course, possible to generalize this approach both to other brain regions and types of information.

As well as assessing the role of rostral temporal areas in semantic representation at the group level, we were also interested in considering the utility of this new approach at the individual level. We found that the group pattern could be observed in the vast majority of individual patients, with some variation in *when* semantically-related patterns were represented in the naming LFPs. This is an encouraging first step and it is possible that the consistency across individual patients may be improved through future studies which further refine data collection and analysis methods.

Finally, we note that the results of the current study add to those that implicate the vATL as an important contributor to language-semantic function (see Introduction). Consistent with the hypothesis posed in a previous study (Shimotake et al., 2015), it would appear that the “basal temporal language area” may reflect its more primary role in representing semantic concepts. This result is consistent with the fact that anomia and mild semantic impairments are observed after resection (Antonucci et al., 2008, Bell et al., 2001, Lambon Ralph et al., 2012) and that, if the region is spared by using a subtemporal surgical approach, verbal memory is significantly better in the chronic phase post surgery (Mikuni et al., 2006).

## Figures and Tables

**Figure:**
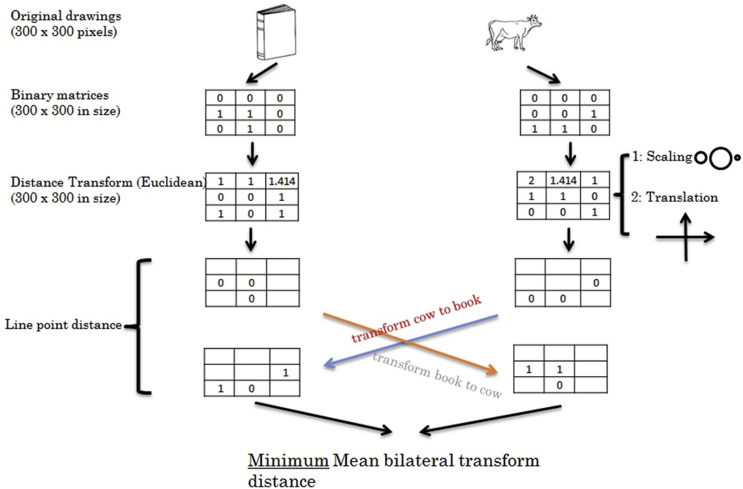


**Table 1:** Patients' demographics and clinical information.

Patient	1	2	3	4	5
Age, gender, handedness	22M R	29M R&L	17F R	38F R	55M R
WAIS-R (VIQ, PIQ, TIQ)	70, 78, 69	72, 78, 72	67, 76, 69	84, 97, 89	105, 99, 103
WMS-R (Verbal, Visual, General, Attention, Delayed recall)	99, 64, 87, 91, 82	99, 92, 97, 87, 83	51, <50, <50, 81, 56	75, 111, 83, 62, 53	71, 117, 84, 109, 72
WAB	95.6	96	97.2	98.5	98
WADA test (Language)	Left	Bilateral	Left	Left	Left
Age of seizure onset	16	10	12	29	55
Seizure type	Non-specific aura → CPS, GTCS	Aura(metamorphosia, epigastric rising sensation) → CPS	Discomfort in throat → CPS	Epigastric rising sensation → CPS	CPS (once)
Ictal ECoG onset	aMTG	PHG	PHG	PHG	none
MRI	L basal frontal cortical dysplasia L anterior temporal arachnoid cyst	L posterior temporal cortical atrophy	L temporal tip arachnoid cyst	L hippocampal atrophy/sclerosis	A low grade glioma in the L medial temporal lobe
Pathology	FCD type IA	FCD type IA Hippocampal sclerosis^a^	FCD type IB	Hippocampal sclerosis^b^	Diffuse astrocytoma

Patient	6	7	8	9	10
Age, gender, handedness	34M L	41F R	27F R	51M R	38F R
WAIS-R (VIQ, PIQ, TIQ)	55, scale out,44	72, 83, 75	106, 102, 105	73, 97, 83	109, 115, 112
WMS-R (Verbal, Visual, General, Attention, Delayed recall)	52, <50, <50, 55, <50	83, 111, 89, 94, 82	112, 114, 114, 81, 100	80, 101, 85, 1919, 91	71, 79, 70, 90, 58
WAB	88	97.3	99.6	89.6	96.9
WADA test (Language)	Left	Right	Left	Left	Left
Age of seizure onset	12	19	16	43	28
Seizure type	Aura(déjà vu, piloerection, auditory aura) → CPS	Aura (nausea, feeling pale) → CPS	Epigastric rising sensation → CPS	CPS	Non-specific aura → CPS
Ictal ECoG onset	R parietal lobe ∼ pMTG	PHG	ventral anterior temporal	mITG	SMG
MRI	R parietal cerebral atrophy & contusion R hippocampal sclerosis/atrophy	L hippocampal atrophy/sclerosis L parieto-occipital perinatal infarction	R mesial temporal cyst	Left temporal cavernoma	L parietal opercurum tumour
Pathology	Traumatic head injury Hippocampal sclerosis^a^	FCD IA Hippocampal sclerosis^a^	FCD IA	Arteriovenous malformation	Oligoastrocytoma

CPS: complex partial seizure, GTCS: generalized tonic clonic seizure, ECoG: electrocorticogram.

a/pMTG: anterior/posterior part of the middle temporal gyrus, PHG: parahippocampal gyrus, FCD: focal cortical dysplasia.

a Dual pathology.

b Diagnosed by clinical findings.

**Table 2:** Brain regions whose activity patterns showed correlation with theoretical models, at least *p* < .01 voxel uncorr, minimum ext = 10, *p < .05 FDR cluster corrected, **p < .05 FWE cluster corrected.

Region	Cluster		x, y, z	*T*	*Z*
**Semantic feature model**					
L Inf temporal	233	**	−33, −9, −48	5.02	3.38
		−33, −15, −42	4.77	3.29
		−51, −24, −36	4.6	3.22
**Semantic feature (visual dissimilarity partialled)**					
L Inf temporal	233	**	−33, −9, −48	5.03	3.38
		−33, −15, −42	4.8	3.3
		−51, −24, −36	4.57	3.2
**Semantic feature (phonological dissimilarity partialled)**					
L Inf temporal	231	**	−33, −9, −48	4.99	3.37
		−33, −15, −42	4.73	3.27
		−51, −24, −36	4.62	3.23
**Visual model**					
L Inf temporal	27		−39, −18, −30	3.86	2.89
		−36, −21, −39	3.38	2.65
**Category model**					
L Inf temporal	274	**	−45, −18, −33	5.79	3.65
		−36, −12, −48	5.38	3.51
		−51, −24, −36	5.25	3.47
