# Supplementary material for: The ‘when’ and ‘where’ of semantic coding in the anterior temporal lobe: Temporal representational similarity analysis of electrocorticogram data
Source: Cortex. 2016 Jun;79:1–13. doi: 10.1016/j.cortex.2016.02.015 (PMC4884671; doi:10.1016/j.cortex.2016.02.015)
Supplement: Supplementary file 1 [file mmc1.docx]

Figure S1: Chamfer-matching computation of visual similarity between stimuli. Each picture was first converted into a binary matrix. A distance transform matrix was computed for each image. This results in the value representing Euclidean distance between each pixel and the nearest non-0 pixel. The transform distance from image A to B was computed by identifying pixels with value of 0 from image A and corresponding position pixels in image B. The sum of the selected pixel values in image B represents the transform from image A to B. The same procedure was repeated to compute the transform distance from image B to A, and the two values averaged. As images can vary arbitrarily in space and size, scaling and translation movement was performed on the distance transform matrix of one of the images iteratively. Global minimum mean bilateral transform distance was used to represent the visual dissimilarity between the pair.

Table S1: Stimuli

bat

cockerel

crab

dragon

ladybird

tortoise

whale

ant

butterfly

bear

bee

camel

cat

caterpillar

cow

deer

dog

duck

eagle

elephant

fish

fly

fox

frog

giraffe

gorilla

goat

horse

kangaroo

lion

lobster

monkey

mouse

ostrich

owl

peacock

penguin

pig

rabbit

raccoon

rhinoceros

seahorse

sheep

snail

snake

spider

squirrel

swan

tiger

zebra

cymbals

shell

ski

slide

tractor

web

yo-yo

aeroplane

anchor

barrel

basket

bell

cake

cannon

chain

church

cigar

clock

crown

dress

drum

fence

flute

glove

guitar

gun

hammer

harp

helicopter

iron

kite

ladder

mitten

motorbike

nut

piano

pram

skirt

sledge

snowman

suitcase

swing

toaster

train

trumpet

vase

violin

wheel

whistle

windmill
